# A Qualitative Study of the Views of Patients With Medically Unexplained Symptoms on The BodyMind Approach^®^: Employing Embodied Methods and Arts Practices for Self-Management

**DOI:** 10.3389/fpsyg.2020.554566

**Published:** 2020-12-07

**Authors:** Helen Payne, Susan Deanie Margaret Brooks

**Affiliations:** School of Education, University of Hertfordshire, Hatfield, United Kingdom

**Keywords:** self management, medically unexplained symptoms, patient perceptions, embodiment, qualitative research, The BodyMind Approach, the arts

## Abstract

The arts provide openings for symbolic expression by engaging the sensory experience in the body they become a source of insight through embodied cognition and emotion, enabling meaning-making, and acting as a catalyst for change. This synthesis of sensation and enactive, embodied expression through movement and the arts is capitalized on in The BodyMind Approach^®^ (TBMA). It is integral to this biopsychosocial, innovative, unique intervention for people suffering medically unexplained symptoms (MUS) applied in primary healthcare. The relevance of embodiment and arts practices in TBMA are discussed in relation to the views of participants in the pursuit of self-management. If widely employed TBMA could have an enormous impact, reach, and significance for patients and global health services. This original pre-clinical trial of qualitative research reports on the perceptions of participant patients with generic MUS, a world-wide issue usually treated by either psychological therapy or physiotherapy. TBMA is not a therapy but a health education program founded upon the concept of an integration of psychological elements with physiological, bodily, and sensory experiences. Thematic analysis of qualitative data sets from open-ended questions in semi-structured interviews and a written questionnaire post intervention is presented. Five aspects which appear to be key to learning self-management were derived from analyzing the data: (1) body with mind connections; (2) importance of facilitation; (3) potential benefits; (4) preparedness for change; (5) self-acceptance/compassion. This article advances the discourse on the nature of self-management for MUS through changing the mind-set and the relationship participants have with their bodily symptom/s through employing embodied methods and arts practices, challenging current, and solely verbal, psychological conceptual frameworks. Rigor lies in the method of data analysis using cross verification of credibility between reported findings and scrutiny by stakeholders. We conclude that facilitated TBMA groups employing embodied methods and arts practices can act as a method for developing the self-management of MUS and improving wellbeing.

## Introduction

This article reports on original, pre-clinical trials of qualitative research. It is based on an analysis of the views of patient-participants, with medically unexplained symptoms (MUS), on an embodied arts-based practice, “The BodyMind Approach^®^” (TBMA). The aims of TBMA are for participants to develop a changed relationship with their MUS and to enable self-management, both unique to TBMA.

Five themes we call key principles were derived from a synthesis of the analysis of data collected from interviews and questionnaires. These principles explain why TBMA, delivered in the English National Health Service (NHS), demonstrates effectiveness in supporting self-management.

The MUS patient population lacks appropriate, accessible, and acceptable interventions (Chew-Graham et al., 2017), physiotherapy (pain management) and/or psychological services (mental health, cognitive behavioral therapy/CBT) being the only choices available. The latter is wholly unacceptable to this population due to their physical experiences in the body which shapes their explanatory model of their condition as being only physical. In addition, there is a greater stigma associated with psychological/mental health generally. TBMA brings both physiological and psychological aspects together in one unique intervention. TBMA for people with MUS has had encouraging outcomes and there is evidence of its acceptability for this hard-to-reach population.

### Medically Unexplained Symptoms

Previously called “psychosomatic conditions,” MUS (or somatic symptom disorder/body distress disorder) is now defined as chronic bodily complaints for which examinations do not show explanatory structural or other specified pathology (Henningsen et al., 2007), for example, chronic fatigue, headache, chronic pain, fibromyalgia, etc. (Department of Health, 2008; Fink and Schroder, 2010). Patients have recurrent or persistent symptoms, or symptom disorders. Patients with chronic symptoms are extremely common in primary (Steinbrecher et al., 2011; Haller et al., 2015) and secondary care (Nimnuan et al., 2001; Burton et al., 2012), and are a costly (Bermingham et al., 2010) worldwide problem.

### Self-Management of Health Conditions

Self-management in healthcare is defined in different ways, incorporating prevention and decline. It aims to increase the capacity for self-regulation monitoring thoughts, sensations, feelings, and behaviors. The impact of self-management groups has the potential to improve health outcomes, such as increases in patient confidence and physical functioning, adherence to treatment/medication, and reduction of anxiety (Challis et al., 2010).

Multiple ways of knowing (Miller and Crabtree, 2005) incorporate a range of activities to engage patients in reflection and self-awareness, memories, body awareness, dance, body maps, improving body confidence and sensitivity, and enhancing self-care. Body stories of health and illness, and the complex relationship of bodies to life histories and context, are through the art process, rather than solely verbal. As Swartz (2012, p. 21) says when referring to this form of health education, “patients challenge their own situated knowledge and transformation becomes possible.” New and different practices, such as dancing together, result in assumptions about their body being questioned. Written reflections in participant’s journals about their changing body experiences help develop insights and connected knowledge with their own and family/culture and collective knowledge. Through becoming more connected with their bodies, they can know the meanings of, and respond more appropriately to, bodily messages of pain etc., for example, not rushing to the general practitioner (GP) or A&E but valuing, recognizing, and regulating emotions, thus benefiting them, and those around them. Furthermore, this helps people to be able to distinguish between the feeling of connection and disconnection with self and others.

Purdy (2010) found self-management reduced unplanned hospital admissions for chronic obstructive pulmonary disease and asthma. Bjørnnes et al. (2018) in a meta-summary of qualitative research of self-management for women with cardiac pain found support for an individualized intervention strategy. This promoted goal setting, action planning, managing physical and emotional responses, and social facilitation.

The BodyMind Approach^®^ satisfies the aforementioned findings through a facilitated group self-management program with individual goal setting and action plans for people with MUS. It emphasizes multiple ways of knowing, social facilitation, and managing physical and emotional responses. Addressing the long-term aim of self-managing symptoms in a sustainable manner reduces the gap between patient needs and funding constraints.

### The BodyMind Approach^®^

The BodyMind Approach^®^ was first researched in 2004 (Payne, 2009a,b; Payne and Stott, 2010) which showed promising outcomes. Further research with larger numbers demonstrated that TBMA increased wellbeing and activity levels, and decreased depression, symptom distress, and anxiety (Payne and Brooks, 2017). The capital B and M in the term “BodyMind” emerged from an analysis of data retrieved 1.5 to 2 hourly interviews with participants in a previous research study (Payne and Stott, 2010). It emphasizes a bottom-up process, although both “body” and “mind” are important to connect. In TBMA, the body is primary, so comes first, connected to the mind (which is not solely the brain; Siegal, 2012). It also counterbalances current trends regarding “mind–body” concepts (top down).

The BodyMind Approach^®^, called “Learning Groups” for patients and GPs, is a biopsychosocial model, i.e., is interdisciplinary, looking at the interconnection between biology, psychology, and socio-environmental factors, all of which play a role in TBMA. It addresses, for example, the stress response (biological), the MUS person’s mind-set (psychological), and the social and the body environment (the group and the symptom respectively).

The BodyMind Approach^®^ has been specifically designed to support patients with MUS. It consists of an embodied, enactivist (Gallagher, 2019) model derived from dance movement psychotherapy (DMP) (Payne, 2006, 2018; Pallaro, 2007) and experimental practices designed to explore the symptom, and any meaning through the media of expressive movement, drama (role play), clay, mark-making, and writing. These practices are conducted as large/small groups, in pairs and individually. There is a facilitator, a specialist in DMP, adult group work, and expressive embodiment models, trained in TBMA, who initiates and coordinates the practices and the interactions. A TBMA manual supports the facilitator with examples of practices, values, attributes, competencies, and mind-set. The content of the TBMA program is described in detail in Payne et al. (2020).

The group in this report is defined as members of the group and their interactions. The group experience is defined as the facilitation and/or content of practices, or both.

### Arts Methods in TBMA^®^

The United Kingdom APPG (2017) encouraged the use of the arts in health. Arts practices are central and integral to TBMA. The methods adopted have been adapted from DMP to suit the MUS population, for example, dancing synchronously together as a group with and without music (Chace, 1975), and authentic movement (Whitehouse, 1999; Adler, 2002; Payne, 2006). Music is used as an emotional induction tool accentuating group expressed movement qualities and emotions or contrasting with them. Group dance, ubiquitous in humans, involving exertive, synchronized, movement to music, is employed. Research demonstrates that there is a link between exertive, synchronous group movement and elevated pain thresholds, even with low exertion tasks. Synchrony and exertion have independent effects on this measure which suggests endorphins have been released which reduce pain (Tarr et al., 2016). It may also play a role in social bonding within the group setting. A mixed-methods study of group DMP by Shim et al. (2017) aimed to test and refine a model of DMP for pain resilience. It found improved resilience, less kinesiophobia (fear of movement frequently found in people suffering from MUS), increased body awareness, reduced pain intensity, mood, and stress, and increased relaxation; 68% of people felt “moderately to a great deal better” post-intervention. Key mechanisms were activating self-agency, connecting to self and others, enhancing emotional intelligence, and reframing. These results have helped to inform the model of TBMA for pain reduction in people with MUS (since many symptoms involve unexplained pain).

Another element borrowed from DMP is mirroring (Eberhard-Kaechele, 2019) which has been shown to foster secure attachment, synchrony, and emotional regulation. Mirroring can become synchronous (Vicaria and Dickens, 2016). Rennung and Göritz (2016) defined this interpersonal synchrony as instances when the movements or sensations of two or more people overlap in time. Studies on interpersonal synchrony using manipulated synchronized movement show it has positive, especially prosocial, outcomes (Rabinowitch and Meltzoff, 2017). Mirroring promotes dyadic resonance, shapes secure attachment experiences, and facilitates integration (Beebe and Lachmann, 1998). Non-verbal components give rise to right hemisphere resonance affecting attachment relationships, regulation, and emotional processing (Schore, 2003). This process of mutual adaptation in mirroring and Chace’s (1975) model for group movement can be described as motion co-regulation for the purpose of creating synchrony (Hart et al., 2014). According to Fogel (1993), co-regulation is an intrinsic element of dyadic interactions. There are two modes of emotional self-regulation: interactive and autoregulation. Mirroring and group synchronous movement supports the former. These findings inform TBMA.

It appears that insecure attachment is frequently found in people with MUS (Adshead and Guthrie, 2015). TBMA has been designed to take account of the adult insecure attachment styles (Payne and Brooks, 2019). Attachment is fundamentally a regulatory theory (Fonagy et al., 2002). Secure attachment involves the capacity to shift between two modes of emotional regulation, depending on context (Schore and Schore, 2006). Group or dyadic practices using expressive movement provide regulatory opportunities for this adaption between the two modes.

The rationale for including mark-making on paper, clay sculpting, painting with fingers/non-writing hand, and journal writing is that they cultivate a non-threatening environment, there being no right or wrong answers, and are inclusive. They stimulate creativity offering symbolic representation of the symptom, thereby encouraging a change in the perception of it, and the participants’ relationship to it, to make meaning. Verbal and non-verbal symbolization narration evolves. This is an experiential means of shifting from a harsh internal mind-set/critic which monitors the threat response, to an internal, benign, self-caring mind-set, and the associated role that compassion (body and self) has on emotional regulation, and threat management. From the symptom feeling like the “enemy” to be “got rid of,” it can change to becoming an “ally” and to be “accepted.” People begin to recognize that it is their perception of their symptom which is mediating their bodily experience and by relating to it differently, they are more able to see ways of managing it. As a result, there is a change in the perception of mind-set, self, and agency. The symptom symbolized by movement, marks on paper, etc., becomes the gateway to self-development and self-management. Arts practices are an ideal vehicle for distancing the symptom from the person, making it safer for exploration.

The BodyMind Approach^®^ with its integrated arts bias involves the social model of health where improvements in social inclusion and cohesion are important indicators. This contrasts with the medical model, employed by pain management and CBT which separates body from mind. In addition, CBT uses solely verbal methods. TBMA contrasts with other body-oriented models (Rohricht et al., 2019) because TBMA, although also working with the body, works with expression through movement and the arts, and has the goal of self-management rather than cure.

The BodyMind Approach^®^ integrates the body and its sensations with movement and the arts to inspire somatic, cognitive, social, imaginative, and emotional intelligence, alongside engaging participants with the themes emerging from their experience of ill-health and the NHS.

Arts activities can involve aesthetic engagement, involvement of the imagination, sensory activation, evocation of emotion, and cognitive stimulation. Depending on its nature, an art activity may also involve social interaction, physical activity, and engagement with themes of health (Fancourt and Finn, 2019, p. 14).

Fancourt and Finn (2019) also claim that psychological, physiological, social, and behavioral elements are all vital to using the arts in healthcare. In TBMA, “psychological” refers to agency, coping, and emotional regulation; “physiological” to less bodily distress from the symptom; “social” to reduced isolation and social support; and “behavioral” to increased physical activity, healthier behaviors, and self-management development.

### The Intervention

Employing a facilitated group, and an embodied, enactive, expressive approach, is novel for MUS. It is not delivered one-to-one, nor as a group for a specific MUS condition as in CBT. Instead, TBMA is delivered in a heterogeneous group with people experiencing a variety of symptoms.

The TBMA specialist facilitator is crucial according to this study, although it is acknowledged that group support is also essential for efficacy, due to the population’s extreme isolation. MUS sufferers feel they are the only one for whom their GP cannot find a diagnosis. They feel alone because friends/family have become bored of hearing about their symptoms, and often have less motivation to be active and to go out, whereas a group promotes more engagement in life. They may feel helpless and hopeless (due to numerous tests and scans which come back normal). Participants are supported in challenging the notion of hopelessness and helplessness giving a sense of agency. The arts are a perfect medium for combating social isolation, developing agency, and group cohesion. A group can generate new experiences and ideas for creativity, support, and learning.

The intervention included 15 small groups delivered over 12 × 2 hourly sessions, over 10 weeks. Individual consultations, before and after the group, took place with the group facilitator. Group numbers varied between 4 and 10.

Groupwork can be challenging for people, especially if already vulnerable, lacking confidence, and highly anxious, often the case for people with MUS. Groups can be destructive, and access problematic, as research has shown (Smokowski et al., 2001). Consequently, strategies are employed before the group commences to support people to engage and arrive at session one. For example, there is an individual intake meeting with the facilitator at the venue in the week before the first session. This explores people’s fears/questions, provides ground rules/information, and clarifies confidentiality. It engages the participant to find rapport/trust with the facilitator. Furthermore, participants only commit to attending six sessions initially, thereafter re-committing to the subsequent six, sustaining engagement more easily. Most participants attended regularly and completed the program (97%). At the end, there was an exit meeting at which face-to-face semi-structured interviews or a participant experience form (PEF) was administered.

## Methodology

### The Research Question

“What are participants” perspectives on their experiences of TBMA?’ was the research question, aiming to establish participants’ experience of TBMA and if any aspects of TBMA were helpful to developing their self-management of symptoms.

This was a qualitative study evaluating post-intervention perceptions from participants. Groups were delivered via different facilitators (*N* = 3) in different geographical areas and at different times. The qualitative findings reported here, derived from these deliveries, are consistent with the quantitative results previously published (Payne and Stott, 2010; Payne and Brooks, 2018) and provide rich data illustrating process and understanding of the experience.

### Data Collection

This research is a synthesis of qualitative data collected from face-to-face semi-structured interviews (which were recorded and transcribed) on participants’ experiences of TBMA post-intervention (*N* = 18), combined with written qualitative data post-intervention from a PEF (*N* = 24) for which there was a 65% (24/37) response rate. The open-ended questions asked in the PEF were also based on the need to understand participants’ experience of TBMA. The rich descriptive narratives describe participants’ lived world experience of TBMA. The two sources of data collection (interviews plus PEF, *N* = 42) provide for a method of cross-checking data to search for regularities and/or differences. The point of combining all perceptions was to create a larger number of perceptions.

### Ethical Approval

This was gained from the local NHS Research Ethics Committee for the data collection 2005–2009 (number 05/Q0201/63). For subsequent data collection (2012–2016), participants gave written consent to feedback/evaluations being used for research and could withdraw at any time without having to provide a reason. All participants agreed the data could be used for research purposes. Permission was gained from the NHS Clinical Commissioning Group to report on data anonymously in published articles. In all cases, participants were assured of anonymity and confidentiality in the reporting without names or pseudonyms, i.e., only location and year of the group deliveries are referred to in participants’ quotations.

### Sample

Participants were drawn from patients suffering from MUS in primary care in two clinical commissioning groups in the East of England. Recruitment for the groups was via GPs and self-referral confirmed by GPs. All participants presented in primary care with MUS for 6 months plus.

#### Gender

Of the 18 participants interviewed, 15 were female and 3 were male. Of those participants who were invited to complete the PEF, 24 were returned out of which only 14 answered the question on gender. The gender mix, 8% male and 92% female, reflects the literature whereby more women than men somatize (Barsky et al., 2001; Sowińska and Czachowski, 2018).

#### Ethnicity

Of those interviewed, 18 reported being white, although not all British. Out of the 24 PEFs returned, 7 answered British white, and 17 did not answer.

#### Age

Adults of all ages are likely to experience MUS. Of those interviewed, ages ranged from 21 to 81 years; of those completing the PEF, the biggest age category was 46–57 years. The youngest was 24 years and the eldest 63. One did not disclose date of birth.

#### Types of Symptoms

There were 12 different symptoms for this data set of 12/18 patients:

1. Fatigue
2. Widespread chronic pain
3. Back pain
4. Left side pain
5. Lower back pain
6. Being cold
7. Movement restriction
8. Muscle hardening
9. Pain
10. Tiredness
11. Irritable bowel syndrome
12. Anxiety

#### Employment Status

There were data for only eight patients with a mixture of unemployed, retired, and employed full or part time. For all deliveries, most patients remained in the same employment. At the outset, 1/8 (12%) of patients were retired, 0/8 (0%) were in full-time and 3/8 (38%) part-time employment, and 4/8 (50%) were unemployed.

#### Educational Background

Patients had a range of educational backgrounds, for example, none, A levels, degree, and post-graduate. Socio-economic groups were not collected.

### Inclusion/Exclusion Criteria

Inclusion:

• 18+ years;
• MUS diagnosis for at least 6 months;
• Frequent general practice (GP) attender (four visits plus per annum);
• Presentation for 6 months plus;
• Co-morbid depression/anxiety;
• Fluent English speaker.

Exclusion criteria:

• Current relevant physical health problems;
• Fewer than four GP consultations in previous year;
• Trauma in previous 6 months;
• Current relevant physical disability;
• Complex bereavement previous 6 months;
• Learning disability;
• Primary diagnosis of psychiatric condition in previous 6 months;
• Current substance misuse;
• A diagnosed eating disorder.

### Recruitment

There were two methods of recruitment. Health professionals referring were aware of the nature of the research, the intervention, and inclusion/exclusion criteria having attended a presentation and/or received a handout. They selected appropriate patients to whom to give a flyer and made a referral on their behalf. A second method was self-referral, for example, from notices in the community/GP surgery. Self-referrers completed a form reflecting inclusion/exclusion criteria and seeking permission to check suitability with GPs.

### Procedures

For recruitment to groups, following referral on a first-come-first-served basis, patients received a leaflet about the learning group and then, if still interested, attended a half-hour screening interview to establish suitability.

### Analysis

The subsequent manual analysis of the PEF open-ended questions and the interview transcriptions resulted in several themes. Participants recounted examples of their experience of TBMA and ways in which they benefited. For the written responses, the authors scrutinized the narratives identifying common themes and/or differences. Themes were derived from a step-by-step process of categorizing quotations which related to specific content, tracking Braun and Clarke’s (2006) approach to data analysis. This involved noting specific passages of text from the transcriptions and comments on the PEF linked by, or contrasting with, a common theme. This allowed the indexing of the text into categories to establish a framework of thematic ideas about the phenomena. By systematically interpreting and coding the textual data, replicable, and valid inferences were able to be made. Braun and Clarke’s (2006) six steps were followed: familiarization with data, generating initial codes, searching for themes among codes, reviewing themes, defining and naming themes, and producing the final report. Five themes were identified and there follows a description of each theme in detail. These themes acted as proxy indicators for self-management.

This study employed a form of cross-verification to check the credibility of the researchers’ interpretation of the data, against the opinions of six different stakeholders engaged with the study (a referring GP, TBMA facilitators, the NHS commissioner, a non-involved researcher-practitioner with similar qualifications/experience, and one of the participants). In qualitative research, truthfulness can be assessed if the reader resonates with the outcomes as believable, consistent, applicable, and useful to readers and other researchers. All stakeholders were invited to comment on the findings. One of the ways of enhancing validity is respondent validation: “a process whereby a researcher provides the people on whom he or she has conducted research with an account of his or her findings [in order to] seek corroboration or otherwise of the account” (Bryman, 2004, p. 274).

From the stakeholders’ point of view, there is some evidence for transferability to other settings. The reflexive translation into other contexts is where researchers/practitioners assess the extent to which findings in one context apply (or are transferable) to other contexts (Schofield, 1993). Hence, we also invited a research-practitioner to read the report and assess the extent to which the intervention, and findings, could be applied to their context of MUS patients in primary care. Their assessment was that TBMA could be applied in such settings with resulting similar outcomes. Reliability (i.e., the consistency with which TBMA groups would produce the same findings) can be shown because the findings were derived from 15 groups of TBMA, with different facilitators, in different geographical regions, and yet were consistent.

## Findings

### Key for Quotations

P = participant, P1 = participant 1, P2 = participant 2 in the same group/the initial letter of the venue used for the group and the number of the group in which the participant participated where relevant, i.e., P (participant) 1 or 2/the venue followed by year in which the group took place.

People enjoyed attending TBMA, and 95% said they would recommend it to family and friends. The attrition rate was only 3% and satisfaction rated good/very good on all aspects. It is noticeable that participants mentioned elements which they took away from the experience of the intervention without commenting directly on the content of TBMA, which involved movement, dance, and art-making practices; a common finding in similar interventions examining participants’ views (Payne, 1986; Payne, 1996; Kaimal et al., 2019).

Categories were repeated, and comments were plentiful showing saturation of patterns and repetitions. These were formed into five key principles presented here with one or two examples of participants’ comments: (1) body with mind connections, (2) the importance of the facilitator, (3) positive benefits, (4) preparedness for change, and (5) self-acceptance/compassion. It is proposed that the interaction between these elements leads to an integration for self-management.

### Body With Mind Connections

Movement and arts practices were designed to help people make connections between body and mind. People said they learned from witnessing others change their relationship with their symptoms/body. The term “BodyMind” derived from an analysis of participants’ comments who, retrospectively, thought the workshop aim was to link the body with the mind—an aim which, they thought, had been met, for example: *“I learned to link my body with my mind”* (P,H3, 2008). This theme continued throughout the analysis, respondents commenting on how the embodied arts practices helped them find a voice and gain insight into their relationship with their body; listen to and accept their energy levels, and use this monitoring to pace themselves, becoming more in touch with their body, *“Able to pace my day*” (P, HH, 2016) and *“Getting down to the root of the problem”* (P2, H, 2016). Links were made between feelings and symptom severity, how mind tuned in with body giving more of an understanding of the relationship between body and mind, for example, they liked that *“the group was different in that they did unusual exercises like dancing, walking, breathing which helped me cope with my symptoms”* (P, H, 2013).

Participants reported listening to warning signals in the body which helped to learn how to *“live with the symptoms more easily”* (P, H3, 2008) as well as to *“learn how to cope with the symptoms”* (P, H3, 2008).

### Importance of the Facilitator

The data analysis led to the conclusion that the facilitator was essential to the process engendering an inclusive and collaborative style, safety, and support, and challenging participants to take risks. Participants commented that the facilitators were very warm and understanding, knowledgeable, patient, and helpful. For example: *“I learnt so much, and the facilitator is brilliant, she is patient, understanding and very knowledgeable”* (P, H4, 2016).

Some participants noted the facilitator created a safe environment, for example, for the changes required for self-management: *“I won’t trust people easily but trusted the facilitator from day one”* (P, L1, 2015); *“Our leader was a great help in bringing about the group to gel from the start”* (P, H, 2013).

They thought the facilitator listened well, facilitated changes, gave freedom of choice/no pushing, and helped people to learn how to open up, for example: *“[the facilitator] enforced or found boundaries, encouraging participants to listen to their inner voices”* (P1, H3, 2008). In addition, they saw facilitators as being approachable, insightful, and giving time to each person to be heard and understood. Some facilitators were also seen as nurturers, for example: *“She was nurturing or trying to caress them in a gentle way of exploring something that’s quite painful for some people”* (P, H4, 2008); *“by providing safety (physically and emotionally)—nurturing the group”* (P, H3, 2008). It can be argued the non-judgmental, nurturing, kind attitudes demonstrated by facilitators helped people to open up to self-compassion which is needed for self-management. The facilitator modeled the practices and joined in to lead dance/movement practices and directed the mark-making for participants to engage with and to link these to their symptom/s. Facilitators then guided discussion about symptoms and meanings derived from the embodied experience or artwork.

Facilitators were seen to help people learn how feelings are generated from themselves as opposed to from others—another aspect required for self-management. Participants explained the relationship with facilitators was profound *“it was a deep relationship”* enabling people to identify and express feelings giving tools to overcome symptoms: *“the fact we were given time to express our feelings was helpful”* (P, L2, 2015); *“positive encouragement and tools and space to overcome symptoms”* (P, H, 2013).

Facilitators helped participants to attribute new meaning to symptoms, i.e., turning bodily symptoms into meaningful information about current emotional states, typically saying: “*Realising my emotional situation is not helping my physical problems”* (P, H, 2015). Recognizing these states, participants were guided by facilitators to inform themselves of their needs and ways to self-manage: *“I learned new ways of coping”* (P, H, 2013). Facilitators convinced people to become interested in the meaning of their symptoms, and the part the symptom plays in their lives, accepting the symptom for what it is without judgment. The reward for learning is the continued capacity for growth, to go forward knowing they can be resilient in the face of adversity when it hits: *“[the facilitator] helped me find a way of moving forward”* (P, HH2, 2016).

Facilitator attributes include the ability to model staying in the present moment (Stern, 2004), for example: *“[the facilitator] helped me be more in the present”* (P, H4, 2008); *“[the facilitator] was excellent and always present”* (P2, L1, 2015); *“learned to be more present with me and symptoms”* (P, H3, 2008) which helped to promote connection with the body and the symptom to bring about self-management. Focusing on bodily sensation aids in the intention to be present, as well as focusing on the arts-making or movement process (as in adaptive authentic movement). Furthermore, creative experimentation with reference to the symptom in a safe environment, for example, creative movement with hands, reflective writing, or mark-making, encourages an intention to stay with the present moment.

Facilitators engender and model non-judgmental and empathic attitudes, picked up by participants, to provide sufficient safety and support to open up to playing, creativity, risk-taking, and exploration, etc. For example, *“I liked fact it is free and non-judgemental”* (P, L1, 2015); *“meeting others who had similar problems and not being judged”* (P, H, 2016).

### Positive Benefits

From the data analysis, it is concluded that positive benefits from the arts practices in TBMA were feelings of (1) belonging, (2) support, (3) safety, and (4) shared purpose all of which are required to help develop the confidence to learn to self-manage. In a group, each person brings potential for rich experience whereby numerous perspectives are available. The experience in the group, and the group itself, was an important vehicle for change for almost all participants, for example: *“[it] worked like a catalyst”* (P, H2, 2008), or as a starting point offering an option: *“This group sort of showed me a road, and I could go down it, or not”* (P, H3, 2008); *“[The] group supported me and helped me to accept the limits of my condition”* (P, L1, 2015).

The experience gave people a sense of belonging: *“Feeling less alone”* (P, H, 2016), support and sharing: *“This course gave me the chance to unite, share experiences and support each other and gave a sense of belonging”* (P, L2, 2015). Discovering others with similar, or other, unexplained conditions helped feelings of belonging. Understanding they have had similar NHS experiences and seeing symptoms from new perspectives bonded people: *“being part of a group with people who are in a similar situation and learning to see your symptoms from a different angle”* (P, L1, 2015). Feelings of isolation were reduced: *“I found getting other people’s feedback and hearing their experiences helpful”* (P, L1, 2015). Participants made friends: *“The group and our leader were all very friendly and helped each other, a good experience, I made a number of new friends, the group really gelled from the start”* (P, H, 2013). Participants mentioned they met group members post course on a voluntary basis: *“I met people with a similar condition to myself and we are still meeting after it all ended”* (P, H, 2015). Seeing how others changed in their relationship to their body helped participants too *“I have gained from the experience, particularly learning about my relationship with my body, and seeing it reflected in the others in the group”* (P, H, 2013).

### Preparedness for Change

Participants appeared to make changes, whereas previously they had resisted this. They reported on changes in their symptoms, lifestyle, and mind-set (thinking) resulting from the experience of engaging with embodied experiments with the arts and creative movement. Such change could form the basis for self-management: *“I achieved a return to work and overcoming of fibromyalgia pains and symptoms in an on-going manner”* (P2, H, 2013).

Changes in thinking and habits developed, for example: *“Changing thinking patterns and habits”* (P, H, 2016), as did changes in lifestyle: *“I was very set in a pattern, and it sort of acted as a catalyst to start my life changing, and it’s changing more now, and quicker”* (P, H2, 2008) and mind-sets—all aiding self-management: *“a shift in outlook, [I have] a more positive approach”* (P, W, 2016); *“facilitated a shift in how to manage life”* (P, H2, 2008).

Hope for the future became important in the embedment and maintenance of new habits, reduced worries, and changed mind-sets for sustaining self-management: *“I have been able to feel less worried and anxious about the future”* (P, S, 2016); *“I feel more positive in my outlook and look forward to my future”* (P, L2, 2015). Mentioned frequently was the phrase*:“I had enough help to go forward”* (P, L1, 2015). Other comments on hope for change included: *“I will find life more enjoyable since being involved with the facilitator and the group”* (P, L1, 2015).

Comments concerning change and choices included: *“I do take breaks and do whatever—silly things, but to give pleasure*… *jogging, listening to music or bubbling around somehow”* (P, H3, 2008); *“I question myself more before I commit myself to do so many things”* (P, H3, 2008).

Some participants referred to the change in reflections on the “self”; these statements concern a deeper knowledge and understanding of self as a pre-requisite to change, for example: *“Taught me a great deal about myself”* (P, L1, 2015); *“Questions I was asked made me look deeper into what was happening to me, making me think deep thoughts I had never realised needed looking into but have helped me very much”* (P, L2, 2015 *“I [learned about] the inner self, to discover what you want and need (and to what extent*)*”* (P, H2, 2008). Other comments were symptom related such as insight into the meaning or cause, developing coping strategies, and learning about their symptoms through the different art forms: *“[I learned] to find out more about the symptoms”* (P, H2, 2008). Flexibility and embracing the possibility of change in their life emerged; some mentioned reduced anxiety/stress: *“improved stress management, therefore the symptom disappeared!”* (P, H4, 2008).

Participants thought the intervention had affected their lives to change both physically, such as feeling more energetic, relaxed, fitter, and in their overall wellbeing: *“My wellbeing has really improved generally”* (P, H1, 2008). Valuing self, indicating improved self-esteem emerged: *“I now value myself and the quality of my life”* (P, H1, 2008) and having more will power and self-reflection. For self-management, there needs to be acknowledgment of physical change as well as self-reflection and motivation to sustain it. Ego strength provides the energy for self-agency and the ability to show it improved: *“I have direction now”* (P, L2, 2015); *“enabled me to help myself”* (P1, H5, 2009).

*[The] activities taught has enabled me to control my anxiety and be more in tune with what my body needs. The many concepts and strategies taught have made me feel more empowered to tackle this illness and I am noticing a direct correlation between the level of anxiety and the severity of pain felt* (P, W, 2016).

With reference to agency, it was not only about managing symptoms but also facilitating a shift in how to manage life and feeling more in control: *“more control of life, more structure/routine e.g. in family life”* (P, H3, 2008); *“It might give you your independence back from seeking medical advice all the time”* (P, H5, 2009). This would suggest that TBMA may save health service resources.

Other changes involving the management of feelings, characterized by comments such as *“I discovered that I get my symptoms through a lack of expressing how I feel”* (P, H4, 2008); *“to understand how worries can trigger symptoms”* (P, H5, 2009) show participants appear to have learned about repression, the influence of feelings on symptoms, and the importance of expression as fostered by the embodied arts practices. Such learning can support strategies for self-management.

Participants found they had changed to be at the start of a journey: *“I feel I am only beginning this journey, as the changes I am putting in place will hopefully have an accumulative effect over time”* (P, H1, 2008). People said they were taking more time for fun things, breaks, changes in diet, and how to improve their stress levels. In terms of the sustainability of changes, a typical comment was: *“[the course] was life changing”* (P, H4, 2008).

Emphasizing the change in positivity more readily appeared from a number of comments: *“friends have commented on how much I have improved with my positivity”* (P, S, 2016) and *“the course brought me to the point of ‘I need to start living again’—it has helped me no end”* (P, W, 2016). The latter suggests life has been on hold due to MUS. Participants appeared to feel more empowered to tackle their illness, to start to do things for themselves such as meditation or painting, were more able to take on new things, and found a way to move forward with self-care and less dependence on the NHS.

### Self-Acceptance/Compassion

Self-acceptance and compassion derived from many comments. The symptom is transformed from being an “enemy” into an “ally” highlighting tolerance and acceptance—part of what is needed for self-management: *“I have become much more tolerant and understanding of my condition”* (P, S, 2016); *“It was a reminder to make time and space for myself, be more accepting and more relaxed”* (P, H, 2013); *“I have learned to accept it is OK to have limitations on what I can do”* (P, L2, 2015)*; “Whilst I know the sessions won’t cure my illness but I have learnt to accept it more, through different ways”* (P, H3, 2016); *“[I] let go of my shame about my condition*. *[I am] in a much more accepting place which will help me achieve my goal of managing my energy better”* (P, L1, 2015). Valuing the self is required for self-management: *“[I have] enhanced self-value, self-confidence”* (P, H1, 2008); *“being kinder to self”* (P, W, 2016); *“learning to try and relax more and think about myself”* (P, H1, 2015).

## Discussion

All these findings represent participants’ take-home experience of the intervention in their terms. The content of sessions was based on arts and embodied practices to exploring the symptom. While participants have not generally reported on these, they were the vehicle for experimentation and exploration to learn more about their symptoms, to provide a platform for self-management.

It is acknowledged that MUS sufferers are hard to reach, perhaps because of the limited treatments acceptable to them; they are hard to define and have varying levels of trust and confidence in the health service. TBMA appears to be a safe and non-threatening pathway to engaging this population.

Mobini (2015, p. 9) states “one of the major obstacles of delivering any psychological treatment to this clinical population is that often psychological treatment is considered as irrelevant and so referral to mental health services as unacceptable” referring to patients’ views. TBMA appears to be seen by participants as relevant, as it is not framed as a psychological treatment but as a course of learning about symptoms and their management.

They may also be hard-to-reach due to the shame attached to being ill and/or sometimes absent from work with symptoms which are unexplained (inferring they are spurious, when they are real), for example, one participant said: *“I learned to let go of shame about my condition”* (P, L1, 2015). Such feelings of shame can be resolved if meaning can be attributed to the symptom, providing for a sense of control.

The themes mentioned demonstrate several important ingredients and inter-relating aspects for self-management. The following discussion for each of the five themes makes links to the literature.

### Body With Mind Connections

The body with mind connection concept is a new and emerging area within the worldwide problem of MUS. In TBMA, the individual is nurtured toward engaging with their body symbolically, to view/experience it not only as a source of pain, discomfort, and negative experiences, but also to acknowledge healthy, functioning aspects. Identifying sensations of symptoms, and where they are in the body in a mindful way, with kind attention, leads to being able to control the distress. TBMA links sensations (interoception) to feelings and the imagination (via arts practices) and perception of the external environment, raising body awareness to find meaning in the symptom.

Seeing others make connections between body and mind and making meaning of their symptoms can stimulate participants to make their own body with mind connections. Making these connections brings an appreciation of the ability to use bodily signals to self-regulate in a positive way, as opposed to previous, rather hostile, perspectives. TBMA promotes inhibition of old habits, reappraisal of pre-existing assumptions, and possibilities to respond to stress in novel ways offering greater emotional self-regulation. Because emotions and movement are so closely related (Kirchhoff, 2018; Melzer et al., 2019), non-verbal behavior, as in expressive movement (Krantz and Pennebaker, 2007) and mark-making practices, can encourage reflections on a range of feelings (sadness, fear, anger, joy) which some people with MUS find difficult to identify and verbalize (alexithymia). This somatic, bottom-up intervention removes the focus on verbal language and memory, working with implicit elements available in the non-verbal. It appears that the creative process, which involves play (Porges, 2015) within the group interactions, can lighten feelings of helplessness and hopelessness (Seligman and Groves, 1970).

Exploring the nature (or purpose) of the symptom, through experimenting with arts practices, creates distance between participant and symptom, allowing safety for meaningful aspects to emerge. Exploring sensorimotor experiences of symptoms, and employing grounding, synchronous movement together with others, mirroring, and centering practices, can help reclaim emotional self-regulation and feelings of safety, leading to a greater sense of control.

### The Importance of the Facilitator

Facilitators were perceived as catalysts, crucial for engendering hope for change. They were seen as knowledgeable specialists, role models, and a safe pair of hands, creating a secure boundaried environment, helping people to open up and feel comfortable, ensuring all were heard and understood. Participants felt safe enough to take risks required to engage with the practices, and express feelings, because group cohesion (Forsyth, 2010) could be relied upon.

Facilitators honored symptoms, accepting them, and the person, unconditionally without judgment or questioning. Participants did not need to disclose their symptoms, but all were invited to bear their symptom in mind when engaging with practices. Symptoms were perceived as acting as agents for participants to listen to their own inner body-felt voices and address their problems. Facilitators valued participants’ lived body experience, helping them to look at it afresh and focus on it as opposed to pushing it away; seeing, feeling, and experiencing it “as is” rather than what they would like it to be, i.e., gone forever.

### Positive Benefits

The benefits were feelings of (1) belonging, (2) support, (3) safety, and (4) shared purpose. These were all present in TBMA groups and seemed important to the efficacy of TBMA according to participants. Cohesion is a general term for assessing the quality of the whole group, based on group integration and individual attraction to the group (MacKenzie, 1997; Dion, 2000). Participation increases in a cohesive group, and in return produces more interactions between members, leading to a more productive and effective group with better outcomes for its members and the group as a whole (Tschuschke and Dies, 1994; Johnson and Johnson, 2000; Marmarosh et al., 2005; Marmarosh and Van Horn, 2010). Cohesion is regarded to be the most important process in a group (Brown and Lent, 2000; Corey, 2004; Delucia-Waack and Bridbord, 2004; Yalom and Leszcz, 2005). In TBMA, cohesion refers to the quality of relationships that develop between group members and the facilitator to promote a sense of “belonging,” reducing isolation (Dirkzwager and Verhaak, 2007).

With reference to “support,” participants commented on the significance of the individual members of the group, many opting to continue to meet voluntarily after the sessions and when the program finished. Most participants commented that the interaction with group members improved wellbeing and gave a sense of support. For example, many arts practices took place individually (as in mark-making), in dyads (as in mirroring), or as a whole group when employing the Chace (1975) model of actively moving in synchrony to music together. After individually being engaged in an arts practice, participants would pair up to share and support one another. On other occasions, participants would offer support to each other in the manner of witnessing, found in authentic movement (Whitehouse, 1999).

In TBMA groups, “safety” is fundamental, and the program is designed with that in mind (Payne and Brooks, 2019) because insecure attachment has been associated with MUS patients (Adshead and Guthrie, 2015). A positive perception of the group can indicate that members view the group as trustworthy and safe to explore and practice new skills (Kivlighan and Tarrant, 2001). It is characterized by participants being active, engaged and seeing the group as beneficial. Orgodinsik and Piper (2003) indicated a correlation between short-term group therapy, climate, and outcomes, whether positive or negative depending on the level of conflict in the group and the phase of the group’s development.

Porges (2003) Polyvagal Theory concludes that human social interaction combined with taking the mind-set into account in interventions turns off the sympathetic nervous system fight/flight/freeze response. The calming of the sympathetic, combined with feeling listened to, enables people to feel “safe” enough to engage in the play required in TBMA arts practices, to do the work of self-reflection to achieve self-regulation and self-management. There appears to be an attitude of kind, loving acceptance in the group experience where all are equal and respected as individuals adding to “safety” and experimentation. This contrasts with their views of previous experiences of unacceptance and disbelief surrounding symptoms. They may have been regarded as psychosomatic, confirming the symptoms are not genuine and, thus, their illness is “illegitimate” (Kirmayer et al., 2004) and/or they should be able to fix it by themselves somehow (Kornelsen et al., 2016).

Participants perceived members as understanding and non-judgmental as people shared experiences of symptoms, and the NHS. They had all joined the program to learn more about their symptoms which already gave a shared purpose. It was never mentioned that the program would cure symptoms, but there was ambition that people could learn to self-manage them. Living with the unknown is extremely stressful; people can begin to imagine the worst, for example, assuming they have the “big C.” They also fear a mental health diagnosis because the assumption is that without a medical diagnosis, all is imagined; one participant said at the first session “*If this group is about mental health then I am leaving now!”* Sharing experiences of symptoms normalizes such fears and the MUS itself.

The experiences promoted change in symptom perception, coping styles, illness beliefs, and personal dynamics—all necessary to achieve an increase in feelings of agency and control for self-management. Survival as a species is dependent on the needs and experiences of others (Beckman and Syme, 1979). Hence, there is dependence upon connecting. We have “evolved the capacity to feel social pains and pleasures, forever linking our well-being to our social connectedness. Infants embody this deep need to stay connected, but it is present through our entire lives” (Lieberman, 2013, p. 10). Furthermore, primates have developed an unparalleled ability to understand the actions and thoughts of those around them, enhancing their ability to stay connected and interact strategically. He goes on to say: “This capacity allows humans to create groups that can implement nearly any idea and to anticipate the needs and wants of those around us, keeping our groups moving smoothly” (p. 10). Although the self may appear to be a mechanism for distinguishing us from others, and perhaps accentuating our selfishness, the self operates as a powerful force for social cohesiveness. “Whereas connection is about our desire to be social, harmonizing refers to the neural adaptations that allow group beliefs and values to influence our own” (p. 11). The embodied, expressive movement experiences in TBMA engender the social connectedness/bonds to facilitate the shared purpose of learning more about symptoms.

The BodyMind Approach^®^ groups share a purpose, practices, values, and beliefs, and these can influence each member of the group positively. We propose the outcome of self-agency, required for self-management, is derived from the social construction developed from confidence gained from the group’s shared purpose, support, sense of belonging, and safety.

Physiologically, although the intervention did not suggest it would cure or reduce symptom distress, many participants commented on how their symptoms had reduced or disappeared. Pain, particularly, was reported to have decreased.

### Preparedness for Change

Cohesion in the group leads to safety and more risk taking in giving feedback and establishing interpersonal relationships (Yalom and Leszcz, 2005). Risk taking leads to change in a group setting (Greer, 2012). TBMA encourages risk taking and therefore opportunities for change in a safe, interactive environment. For example, self-disclosure, feedback, facilitator, and group members’ contributions all reflect risk taking and increase cohesion in the group (Burlingame et al., 2011). Research suggests cohesion plays a role in the outcomes of groups (Brown and Lent, 2000). In TBMA, there was a shift, according to participants, to becoming more positive for hope for change for the better and a belief that change was possible (i.e., to self-manage symptoms). Kivlighan et al. (2016) found a significant relationship between an individual member’s post-treatment hope and the aggregated sense of hope of other group members. Participants reported new habits, feelings, routines, lifestyle, and mind-set (Wood and Runger, 2016)—all required for self-management. Self-understanding and making meaning appeared to be pre-requisites when making changes.

Participants described the group as life-changing, such as helping reduce anxiety about the future, improving stress management, and regaining interest in past pursuits. Participants reported improved physical and mental wellbeing enhancing self-esteem, willpower, and self-regulation, pacing themselves better and cultivating healthier routines. Recognizing feelings and understanding their relationship with symptoms and triggers for their symptoms and emotions were important in the group. Links were made between thoughts, feelings, and the sensation of symptoms.

Change was described as the beginning of a journey, and the belief expressed that the improvement can accumulate over time, with on-going conscious and/or unconscious impact. Some people felt empowered to tackle their illness noticing a relationship between anxiety and pain. la Cour and Petersen (2015) found an association between anxiety and pain relief. All this points to the capacity to learn hope for the future and take some control, as opposed to their previous state of learned helplessness (Seligman and Groves, 1970).

Role play (Corsini, 2017) is both part of the group experience and a practice in TBMA, for example, asking participants to share in pairs how their symptom may move or the posture it might take up. This often leads to greater insight into the meaning of symptoms. The languaging of the feeling state in such a posture, for example, might contribute to a greater meaning-making of its nature and role in life. Feedback received from their partner could add further to this meaning. For example, a participant saw the image of a lion emerge from sensing her symptom through non-directed, expressive bodily movement, which she interpreted as anger. During the verbal dyad process, her partner also saw features of this animal. The dialogue that followed between them helped this participant to consider how to moderate her anger which she realized tended to trigger her symptoms. The participant dialogued with their symptoms to explore and better understand, re-frame, or gain an explanation of meaning, origins, triggers, and maintenance of them day to day.

Confidence gained in the group enabled the reduction of shame and improved wellbeing, self-esteem, and the capacity to believe things could change for the better, promoting the development of a mind-set to embrace change and self-management skills.

### Self-Acceptance/Compassion

Through the accepting ethos offered by TBMA, participants are afforded the opportunity to learn the value of self and others. Self-acceptance/compassion was concerned with understanding other participants’ conditions and having a tolerance of them, with kindness. For example, the notion of accepting the symptom, non-judgmentally, appeared often. Being more connected to their body appeared to allow a greater capacity to notice energy limits and for when to rest/relax. Sharing experiences in the group helped participants gain a perspective of their own situation. Journaling provided enforced time to reflect on experiences each session. Participants learned, for example, it was acceptable to have limitations allowing them to accept what their body tells them it needs. Comments on outcomes included enhanced self-value and self-confidence changing the mind-set to one of self-compassion.

The honesty encouraged by the facilitator in the group nurtures people to accept the idea they have a condition which, although may not be curable, is something they can learn to manage. It should be noted in some cases symptoms did disappear. Finally, acceptance (of their condition) in the supportive group and self-acceptance/compassion appear to be key to starting to self-manage (Gregg et al., 2007), as opposed to seeking a cure, from the health service. The group promoted greater understanding of the condition and the acceptance of the lack of a cure, helps with an explanatory model which can kick-start change, and obviate the need for more tests and scans. Learning through the group to become kinder to the self reflects an important attitude to developing self-management. Acceptance of self, own needs, and symptoms are a pre-requisite for self-managing symptoms changing the mind-set from “I am not OK” to “I am OK” (Harris, 1970) despite symptoms and limitations.

The changes made to TBMA over the years were only structural, for example, omitting assessments halfway through the program and front loading sessions to twice weekly for the first 2 weeks to facilitate group cohesion and engagement. The reason for few changes is because TBMA is not a prescriptive model, setting out tasks per session as with other structured programs. Trained facilitators will modify TBMA interventions to the group needs, but, within a known framework with topics to be covered, the tailoring of which is down to the professional judgment of the facilitator, i.e., TBMA, responds to the group rather than the group having to adhere to it. There is a manual, and facilitator training is standardized in that it was delivered by the same trainers, and with the same content, adjusted as would be expected, depending on trainees’ needs. There is an assessment process for certification.

After the first 2 weeks, the groups became closed to new participants and remained constant. Group size appears to have been relatively unimportant, probably because the facilitator was responsive to the needs of each group, irrespective of size. The smaller groups did miss those absent at times; there was some depletion of the richness because of this.

We employed dance movement psychotherapists rather than other professional psychotherapists because they would have knowledge, skills, and experience of body-centered exercises and understand the body with mind interdependency. Although TBMA does employ elements from all the arts, specialists in other therapies may have different skills, for example, music, talking, or art therapists.

Potential limitations were the small numbers of participants, the limited amount interviewed (*N* = 20), all groups conducted in one south of England middle-class area, and the need for more trained facilitators and groups. Funding was also limited.

Recommendations for further research would include addressing the aforementioned limitations in a larger qualitative study, in addition to a quantitative or mixed methodology study, further to Payne and Brooks (2017), to include a control cohort, and conducted in more than one region of England.

## Conclusion

This was a qualitative study to discover participants’ perceptions of the experience of TBMA.

We were not evaluating in the quantitative paradigm to discover whether outcomes were as a result of the group or arts experience; therefore, no control group was required. The research was based on an analysis of qualitative data and notes five consistent themes we call key principles: body with mind connections; the importance of facilitator; positive benefits; preparedness change; acceptance/compassion. These all appear to have kickstarted the self-management process for participants.

These five key principles in TBMA demonstrate several important ingredients and inter-relating aspects for the self-management of MUS. It is possible to distinguish the success of TBMA from the influence of the facilitator because there were several different facilitators, delivering in different regions, at different times, with different groups of people and yet all groups demonstrated similar outcomes. The perceptions arose from open-ended questions about the program which did not focus on the arts practices *per se*, independent of the group and the group experience. However, because the arts practices were integral to the group experience, it is reasonable to infer the perceptions reported connected to these practices. It seems likely, therefore, due to the holistic, integrative nature of TBMA, that all these elements may be involved.

We have outlined a model highlighting the interaction between the five key principles in TBMA. It shows how TBMA, with its emphasis on the arts, bodily sensations, creativity, and expressive, movement-based embodied contemplative practices, in a facilitated group setting can help to re-establish sensorimotor integration. Furthermore, TBMA can foster non-judgmental, compassionate, and accepting attitudes leading toward a reconnection with bodily sensations, improved self and body confidence, functionality, and quality of life. These findings expand the scope of reflection regarding the relationship between the arts, body awareness, and MUS, including interoceptive and emotional aspects of the MUS body–mind relationship. It takes further the idea of self-management from its employment with identified diseases to its adoption as a strategy for management/treatment of MUS, which currently have poor outcomes. TBMA may be transferable to other long-term conditions in health education settings.

The potential benefits of TBMA to support patients with MUS to learn to self-manage could be that it replaces current treatments (which are unacceptable to patients) and integrates the body and mind in one holistic biopsychosocial model. Furthermore, it could reduce the high costs for MUS conditions, freeing up GP time, and increasing capacity and resources. The program is unique in its potential reach, significance, and impact because MUS is ubiquitous.

This study is relevant to healthcare as well as to DMP. Benefits from participation in TBMA were feelings of belonging, support, safety, and shared purpose, all important for people in distress. Facilitated group experience is a vehicle for change, and the use of TBMA makes it even stronger.

## Data Availability Statement

The raw data supporting the conclusions of this article will be made available by the authors, without undue reservation.

## Ethics Statement

The studies involving human participants were reviewed and approved by the University of Hertfordshire and NHS ethical approval. The patients/participants provided their written informed consent to participate in this study.

## Author Contributions

HP designed the study and collected the data. SB participated fully in the analysis of the data and the writing of the manuscript. Both authors contributed to the article and approved the submitted version.

## Conflict of Interest

The authors declare that the research was conducted in the absence of any commercial or financial relationships that could be construed as a potential conflict of interest.
